# Mental health self-efficacy as a moderator between the relationship of emotional exhaustion and knowledge hiding: Evidence from music educational students

**DOI:** 10.3389/fpsyg.2022.979037

**Published:** 2022-09-09

**Authors:** Xuan Zhou

**Affiliations:** School of Music and Dance, Xihua University, Chengdu, Sichuan, China

**Keywords:** mental health self-efficacy, emotional exhaustion, knowledge hiding, interpersonal distrust, organizational culture

## Abstract

The knowledge and skills of employees could play a valuable role in organizational success. Organizations seek practices to create a knowledge-sharing culture to take full advantage of individual competencies. However, the knowledge-hiding behavior of individuals is a hurdle in the internal dissemination of knowledge and expertise. It becomes more critical in the case of teaching institutions, where the students are taught and trained. Scholars are now putting their efforts into seeking the antecedents and consequences of knowledge-hiding behavior. This study also attempts to determine the role of interpersonal distrust as an antecedent of knowledge hiding behavior of music education students. Based on the social exchange theory, the present study attempts to check the association of interpersonal distrust with emotional exhaustion and knowledge hiding. For empirical investigation, this study assumes that interpersonal distrust positively enhances knowledge hiding and emotional exhaustion, respectively. Moreover, the present study also attempts to check the association of emotional exhaustion with knowledge hiding. This study also assessed the mediating role of emotional exhaustion in the relationship between interpersonal distrust and knowledge hiding. This current study also aims to check the moderating role of mental health self-efficacy in the relationship between emotional exhaustion and knowledge hiding. For empirical investigation, the present study collected the data from 310 music learning students of various Chinese universities through a structured questionnaire method using a convenient sampling technique. This study applied partial least square structural equation modeling for empirical analyses using Smart PLS software. The findings of this study revealed that interpersonal distrust does not directly influence knowledge hiding; however, interpersonal distrust has a positive association with emotional exhaustion. The findings also acknowledged that emotional exhaustion positively correlates with knowledge hiding. The results also confirmed that emotional exhaustion positively mediates the relationship between interpersonal distrust and knowledge hiding. Further, the outcomes depicted that mental health self-efficacy negatively moderates the relationship between emotional exhaustion and knowledge hiding. In addition, this study’s findings also serve the literature of knowledge hiding by providing important theoretical and practical implications.

## Introduction

The best creative work environment is a stress-free environment that does not deplete workers’ emotional resources. Exhaustion and stress dampen creativity. A stressful workplace discourages employees to work efficiently. Much research has been dedicated to exploring the factors that foster a creative environment; however, there is not much clarity regarding which factors impede employees’ creativity. Therefore, this particular study intends to focus on how social relationships hamper employee’ creativity. Social exchange theory provides the theoretical foundation for the argument presented in this research. This theory explains social relationships based on interpersonal trust. Social relationship in the workplace is negatively affected by interpersonal distrust. Interpersonal distrust invites many troublesome workplace scenarios directly associated with a workplace’s creative output. First of all, cooperation among workers grows bleaker in the wake of interpersonal distrust. Lack of cooperation makes a workplace blunt with less scope for creative endeavors. Furthermore, distrust encourages a culture of knowledge hiding that further weakens the prospects of creativity. An overall negative outlook of the workplace causes an array of untoward outcomes when an organization’s members cease to cooperate hide knowledge and distrust fellow employees (Connelly et al., 2012). Emotional exhaustion results from the depleted emotional and physical resources of an individual. The nature of interpersonal relations determines how emotionally exhausted a target individual can become. However, it is also important to note here that interpersonal relations are the not the only factor that may be held responsible for the emotional exhaustion of an employee rather the factor such as self-efficacy, personal traits and personality characteristics are also of profound importance in this regard (Maslach et al., 2001).

Knowledge hiding is perceived as a negative behavioral trait that occurs when an employee deliberately seeks to withhold valuable knowledge or information to be shared with his/her coworkers even if it is requested (Connelly et al., 2012). Frequent occurrences of knowledge hiding have become a norm in the culture of modern-day organizations and sometimes this negative practice leads to devastating consequences. In order to overcome these negative consequences self-efficacy acts as a moderator. Someone’s self-efficacy is defined by that person’s beliefs regarding his/her abilities to carry out assigned duties in order to achieve the required milestones (Bandura, 1977). According to Bandura self-efficacy has three different roles, such as constructive, predictive and mediational role. These roles have been extensively supported by the findings of many other studies. Behavioral outcomes are more accurately and consistently predicted on the basis of self-efficacy. The current study explores the relationship between interpersonal distrust, emotional exhaustion and knowledge hiding in the educational music students. The theoretical underpinning of this study is based on social exchange theory. Scientists and researchers have studied various aspects of these behaviors but still a study gap exists due to the study’s selection of subject and methodology. This current study is unique in the selection of the subject of research. Up to the knowledge of the author, no study has been done on the music students taking these parameters into consideration. The objective of this study is to explore the already existing scientific data from the available literature to build a foundation for the hypotheses of this study. Moreover, the study aims to alleviate the negative behaviors like knowledge hiding, interpersonal distrust and emotional exhaustion by throwing light on the importance of the use of mental health self-efficacy. It explains the moderating role of emotional exhaustion and self-efficacy and highlights the relationship between interpersonal distrust and knowledge hiding and emotional exhaustion.

The study perceives that mental health self-efficacy negatively moderates the relationship between emotional exhaustion and knowledge hiding. It also assumes that emotional exhaustion positively mediates the relationship between interpersonal distrust and knowledge hiding. The relationship between interpersonal distrust, emotional exhaustion and knowledge hiding is explained by the theoretical framework of the study. The current study finds its basis in the theory of social exchange, according to which human beings are engaged in exchange of relationships based on interpersonal trust. This exchange is reciprocated by the people living in a society, community and organization. Developing an understanding of the concepts of mental health self-efficacy has both theoretical and practical implications. The implications of this study start from merely raising awareness on the given topic of this study and ends with the designing and implementation of awareness and training programs to use self-efficacy to mitigate the negative behaviors like knowledge hiding, interpersonal distrust and emotional exhaustion.

The rest of the article has the following assembly; the review of available scientific literature to design hypotheses of this study, followed by an analysis of data and interpretation of results. The article concludes with a discussion, implications and limitations.

## Literature review and hypotheses development

### Theoretical support

According to the theory of social exchange, human beings are motivated to engage and reciprocate in a social exchange relationship in which the success of the exchange heavily depends on the level of interpersonal trust (Wang et al., 2019). Conflicts arise and interpersonal distrust prevails in politicized organizations where individuals operate in a self-centered manner while ignoring the interests of others (Malik et al., 2019). In such politicized organizations, distrust undermines social exchange relationships in a work environment (Scott et al., 2013). As a result, an employee who does not trust some fellow employee remains unwilling to interact with them and does not maintain a relationship creating an environment of negative interactions (Zhang and Dai, 2015). Many people at such workplaces suffer from workplace ostracism.

### Interpersonal distrust: An overview

Interpersonal distrust is considered as having a feeling of low confidence in others. One perceives that others may act to harm them. Such a person remains wary of the perception that their wellbeing will not be taken care of by others and that others may act in a harmful manner or with hostility. Organizational citizenship behavior may get negatively impacted because of interpersonal distrust and that can increase the tendency of negative outcomes in a workplace (Guo et al., 2017). Interpersonal distrust has a mediating role in the relationship between workplace ostracism and target mistreatment (Scott et al., 2013). The study has also found a deep link between creativity and distrust (Mayer and Mussweiler, 2011). A workplace that has an environment of interpersonal distrust remains stagnant from the productivity point of view. Employees deployed at such workplace remain highly demotivated to interact or establish a healthy working relationship with fellow employees (Scott et al., 2013).

### Relationship of interpersonal distrust with knowledge hiding

Knowledge hiding is considered a negative behavior for its several deleterious outcomes; however, it remains prevalent in almost all the workplaces (Peng, 2013). An outright consequence of knowledge hiding is an ineffective flow of information that has drastic implications for an organization (Černe et al., 2014), furthermore, the social exchange also remains low in such a workplace (Brandts and Solà, 2001). Employees who do not become part of the knowledge-sharing network ultimately remain deprived of the opportunities to enhance their knowledge (Perry-Smith, 2006). It creates more labor for employees who tend to gain knowledge by themselves as they are excluded from the social exchange and knowledge-sharing channels by their fellow employees based on distrust or poor interpersonal relations (Mudambi and Navarra, 2004; Connelly et al., 2012, 2019). Knowledge hiding discourages individuals to acquire already available knowledge or information that ultimately hampers their ability to develop creative ideas (Reiter-Palmon and Illies, 2004). Quite a few studies have established that knowledge hiding has severe implications for collective creativity in a workplace (Jahanzeb et al., 2019; Malik et al., 2019).

Social undermining is referred to as a negative behavioral trait that is exhibited by some toward target individuals in a workplace setting (Duffy et al., 2002). This deteriorates the creative ability of those target individuals and discourages them to have constructive social interaction with other. Target individuals tend to develop a sense of interpersonal distrust vis-à-vis their perpetrators because as mentioned above the process of social exchange is based on reciprocity (Blau, 2017). As per the theory of social exchange, uncertainty and disbelief result from interpersonal distrust (Molm et al., 2000; Blau, 2017). As reciprocity is a vital factor at play in interpersonal relations, therefore, negativity is reciprocated as well. Mistreatment, for instance, in the shape of knowledge hiding encourages the target individuals to distrust the ones who hide it (Černe et al., 2014). Even if distrust is brewing between two individuals that may affect other individuals as well in many different ways. The instinct of knowledge hiding has its roots in distrust as knowledge is hidden from the target individual either as an act of retaliation or an act of punishment (Yuan et al., 2020). There is no denying that knowledge is highly valuable, therefore, it can help one attain a competitive edge over others. Ideally, such knowledge should flow seamlessly in a workplace setting that has a healthy environment enabled by strong social exchange and an absence of distrust. However, interpersonal distrust disrupts the flow of knowledge and dents the network of social exchange (Blau, 2017). Therefore, the motivation for hiding knowledge in a knowledge-based economy is to deprive others of the opportunity to excel or develop using the valuable knowledge to their advantage. Empirical studies have suggested that interpersonal distrust encourages knowledge hiding at work (Yuan et al., 2020). Moreover, it has also been found that knowledge hiding harms the creative output of the target individuals (Bogilović et al., 2017). Therefore, the study hypothesizes that relationship between employee creativity and social undermining can be described through knowledge hiding and interpersonal distrust.

*H1*: Interpersonal distrust positively enhances knowledge hiding.

### Emotional exhaustion: An overview

Emotional, mental, and physical exhaustion caused by prolonged stress is called burnout (Freudenberger, 1975). Frequent occurrence of emotional exhaustion and cynicism in individuals involved in ‘people-work’ of any sort is another way to describe burnout (Maslach and Jackson, 1981). Other than Maslach, researchers believe that job burnout can simply be explained by only one common experience and that is exhaustion (Malach-Pines, 2005). Work burnout remains a challenge for an organization for its high cost in terms of poor outcomes (Halbesleben and Buckley, 2004). It is a negative emotional reaction that causes by excessive and prolonged work-related stress (Maslach and Jackson, 1984; Maslach et al., 2001). Exhaustion and emotional fatigue that are caused by burnout lead to absenteeism, high turnover and poor execution of tasks at work (Wright and Cropanzano, 1998; Grandey et al., 2004). Decreased levels of performance and risk of mental/physical wellbeing increase the importance of tackling burnout issues in the workplace (Taris, 2006). The theory of Conservation of Resources (COR) contends that the pressure of performing well while having limited resources causes burnout. Furthermore, working with limited resources put pressure on the available resources which leads to their poor utilization (Hobfoll, 1989). The extent to which the employees can engage in regulating their emotions depends on the physiological arousal of stress-inducing factors (Gross and Levenson, 1997; Brotheridge and Grandey, 2002; Butler et al., 2003). The extent of this emotional regulation manifests in the shape of poor performance at work as well as burnout (Brotheridge and Grandey, 2002). The risk of burnout increases manifolds in the wake of prolonged and chronic work-related stress. This risk compounds in the presence of a combination of chronic job stress, distrust among employees, poor social exchange and daunting demands of productivity (Schmitz et al., 2000). Many studies have shed light on the fact that differences among individuals have a direct impact on the extent of burnout that workers have to endure during their course of work. Many systematic reviews and meta-analytical researches have identified certain individual characteristics such as personality traits and personal experiences responsible for work-related burnout (Alarcon et al., 2009; Brown, 2012; Lee et al., 2013).

### Relationship between emotional exhaustion, interpersonal distrust and knowledge hiding

Conservation of resource theory (COR) describes the link between emotional exhaustion and knowledge hiding (Hobfoll, 1989). The COR theory asserts that there are many different types of resources that people possess that they look to preserve, utilize, maintain and attain to achieve their targets (Hobfoll, 1989). The work-related strain has a variety of different strains, however, emotional exhaustion remains one of the most extreme varieties. Employees experiencing emotional exhaustion generally have damp feelings, low trust, shallow interest and deflated level of motivation (Maslach and Jackson, 1981). Emotional exhaustion also leads to frustration, fatigue and irritability (Maslach and Jackson, 1984). Employees feel unable to upkeep a stable psychological state in the wake of depleted emotional resources. There is a sequential process of burnout, as it is proposed, that involves emotional exhaustion, client depersonalization and undermining of one’s achievements. Thus, using the above scientific evidences, it is hypothesized that:

*H2*: Interpersonal distrust positively enhances emotional exhaustion.

*H3*: Emotional Exhaustion positively enhances knowledge hiding.

### The mediating role of emotional exhaustion

Emotional exhaustion subsides in a workplace that is guided by a supportive leader and has a cohesive work environment where people find it comfortable and encouraging to share their experiences in a mutually benefiting way (Freudenberger, 1975). Maslach and Pines found that frequent meetings held at the workplace are beneficial for reducing emotional exhaustion especially if the employees share their troubles and seek to either provide help or ask for assistance (Malach-Pines, 2005). There is a negative correlation between job feedback and emotional exhaustion according to a study carried out by Maslach and Jackson (1984). The scientific studies have shown that burn out and emotional exhaustion makes negative outcomes in the personality and behaviors of the individuals. These negative outcomes include interpersonal distrust and knowledge hiding. So this study develops another hypothesis based on the scientific evidence. The hypothesis states the mediating effect of emotional exhaustion on the relationship between interpersonal distrust and knowledge hiding.

*H4*: Emotional exhaustion positively mediates the relationship between interpersonal distrust and knowledge hiding.

### Mental health self-efficacy as moderator between emotional exhaustion and knowledge hiding

Self-efficacy is both the belief in one’s abilities to execute the job by executing a particular course of action demanded by a situation (Bandura, 1977) and the ability to make a variety of judgments that are required by the job at hand to successfully achieve what is required of them to perform (Riggs and Knight, 1994). The workers with a high level of self-efficacy have a decreased level of job stress in the wake of work control and autonomy.

Workers with a high level of self-efficacy tend to better deal with and master the stressors as compared to others whose self-efficacy level is low. There are multiple levels of self-efficacy. These levels are associated with different behavioral reactions of employees in response to a particular situation. Employee’s reactions and behavioral tendencies are directly associated with their level of self-efficacy. This sense of self-efficacy helps employees overcome job-related stresses and barriers. It has been found in many studies that a person’s perception regarding their social capital such as the level of self-efficacy and autonomy at the job, helps reduce the emotional labor and job-related stress (Heuven et al., 2006).

Four techniques can enhance the self-efficacy of an individual. These techniques are as follows: verbal persuasion, enacting mastery, role modeling and affective arousal. It is an assertion of social learning theory (Bandura, 1977) that learning can be enhanced through paying focused attention to the attitudes, values and behaviors of role models (Brown and Treviño, 2006). The theory suggests that deep awareness of desirable and undesirable behaviors can be achieved through paying keen attention to the modus operandi of role models.

Individuals possessing a sense of strong self-efficacy are better equipped mentally to cope with stressful situations without getting fussy or stressed. This particular ability to remain calm under stressful situations is a manifestation of the belief in one’s own ability to handle a particularly stressful situation (Bandura et al., 1999). It has been observed that perceived self-efficacy predicts stress levels and anxiety that are manifested and faced in interpersonal transactions (Alden, 1986). Vulnerability to burnout surges in the exposure to chronic stressors particularly if the level of self-efficacy is low along with high pressure to perform well at the job (Schmitz, 2008). When the stress is unmediated, the load of work is overwhelming, support is non-existent and safety buffers are not present, burnout remains a highly likely outcome. If there is a perception regarding a discrepancy between ideal professional performance and the actual result that causes burnout in individuals. A variety of physical and emotional reactions can be expected from an individual going through a burnout phase. These reactions can emotionally be depleting and exhausting and service recipients can be depersonalized by such individuals. Burnout individuals also have a deep sense of personal unaccomplishment (Ropes and de Boer, 2021).

A conceptual model of teacher’s self-efficacy named ‘Classroom and School Context’ (CSC) was devised by Friedman and Kass (2002). According to this conceptualized model, there are two basic domains of a teacher’s functioning at a school. The first domain is that of a classroom where the teacher teaches students. The second domain is that of the school itself where the teacher has to act as a member of the faculty at an educational organization. Both of these domains require a teacher to engage in interpersonal interactions to perform all the professional tasks. The model describes a general list of professional duties that a teacher needs to perform, moreover, the model also describes the contacts that the teacher has to make with other staff members according to his/her self-efficacy. The model suggests that the sense of self-efficacy of the teachers accommodates both these domains that the teacher has to manage at his workplace (Friedman and Kass, 2002). Work-related stress can be moderated through self-efficacy, therefore, a low level of self-efficacy can lead to a higher level of stress (Grau et al., 2001). It was found that professional self-efficacy was positively associated with personal achievements, whereas, it was negatively associated with emotional exhaustion and depersonalization. A certain difference was observed, for instance, in the case of lower generalized self-efficacy emotional exhaustion was found to be higher, whereas, in professional scenarios, a perceived low professional self-efficacy was found to be associated with higher cynicism and weak organizational commitment. The workers with a high level of self-efficacy were found to be less stressed at work. Studies assert that generalized self-efficacy strongly moderates the relationship between burnout and stressors (Grau et al., 2001).

There are four sources that people use to form their perception of self-efficacy. One of the most influential sources remains the obvious result of one’s performance. If the outcomes are termed as successful the level of self-efficacy raises, however, it lowers in case interpretation suggests the outcomes as a failure. The second source of self-efficacy remains the vicarious experience of the individual when others are seen performing their tasks. This observation enables one to compare and perceive a certain level of self-efficacy. Verbal and social feedback in the shape of persuasions also remains one of the sources that help an individual have a sense of self-efficacy. Self-belief is derived from positive persuasions. Negative persuasions have the opposite impact as it dents and self-belief. Physiological states also suggest the level of self-efficacy as one has to deal with anxiety and stressful instances and certain reactions reflect the level of self-efficacy in an individual.

Mental health self-efficacy can be used as a powerful tool to manage emotional exhaustion and its outcomes like knowledge hiding and interpersonal distrust. Thus from the above discussion, it is hypothesized that mental health self-efficacy acts as a moderator between emotional exhaustion and knowledge hiding.

*H5*: Mental health self-efficacy negatively moderates the relationship between emotional exhaustion and knowledge hiding

**Figure 1:**
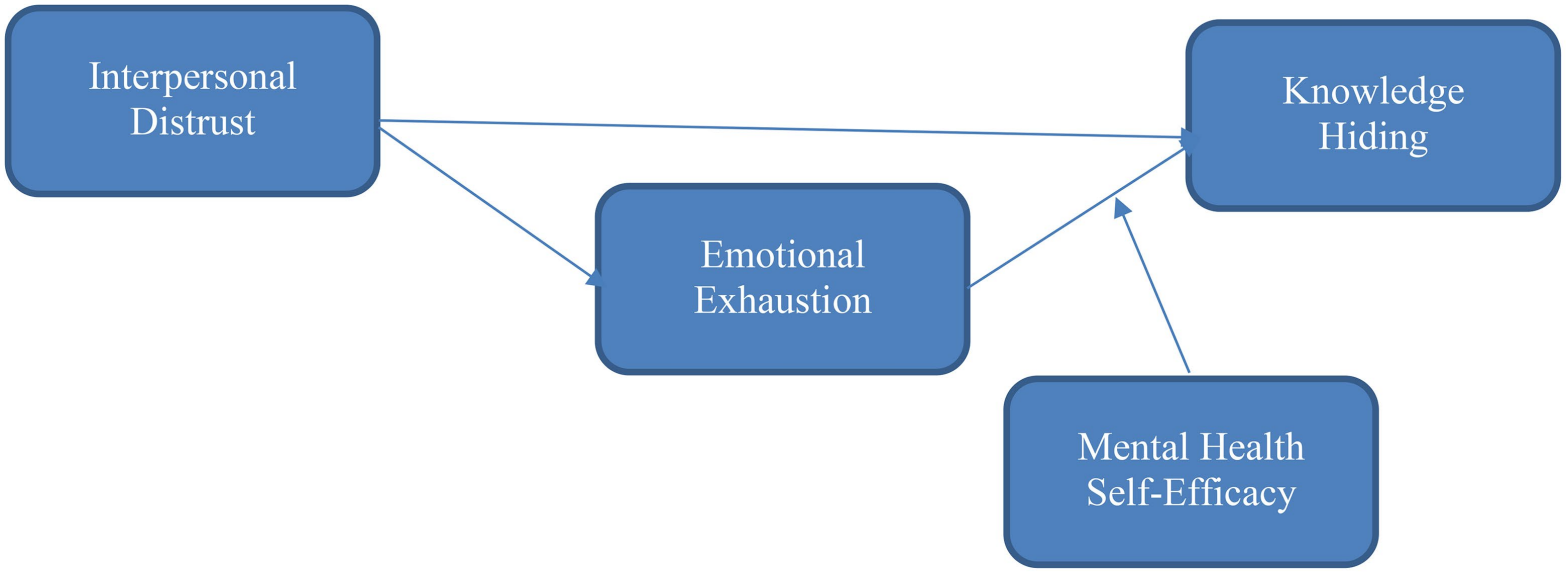
Conceptual framework.

The present study’s conceptual framework is given in Figure 1.

## Research methods

### Study design

This study adopted a convenient sampling technique for data collection. The targeted population of the present study is music learning students of different universities in China. The author visited various universities in this regard and met with the music department heads. In the meeting, the author explained to them the objective of data collection, such as the purpose behind data collection is based on academic purposes. The author also highlights the fundamental practical implications of the present study to heads of the music department. This way, the music department heads permitted the author to gather student data. The author developed a cover letter along with the questionnaire. The purpose of creating a cover letter is to convey the present study’s objective to the students and ensure their data confidentiality. For instance, data would be analyzed only for academic purposes, and aggregated results would be shown instead of individual-level outcomes as individual-level responses would be discarded. The cover letter also confident the students about answering the questionnaire, such as no answers are wrong or right; their actual response would be considered appropriate for the present study outcomes. Hence, students are guided not to consult their answers with their fellow while answering the questionnaires. This way, the students’ confidence was boosted, and they filled the questionnaires with their consent and without any social pressure.

The author also developed dual-language questionnaires to ensure easiness for the students. The author developed the questionnaires in English and then translated them into Chinese. For translation, the author hired an expert in the Chinese language. The author also approved the final translated questionnaires from the senior researchers. As per senior researchers’ guidance, the author also accumulated sample data to verify the language understanding. This way, the questionnaire was finalized to gather data from students. The author also implemented a strategy to reduce common method bias. Such as, data were collected from students under the time lag data approach. Under this approach, the data of different constructs were collected at different times by creating gaps. Hence, it is essential to recognize the same respondents at different times responses. So the author included a hidden code in questionnaires to identify the same student response at different times as the data of the present study was gathered in four turns after one-month gaps between each turn.

For the first time, the author accumulated data regarding the independent interpersonal trust construct. For this purpose, the author distributed 700 questionnaires among music learning students and received 576 complete and valid responses. After the one-month gap, the author distributed questionnaires regarding the emotional exhaustion construct and received 490 complete and accurate answers. Similarly, the third time, the author distributed questionnaires regarding knowledge hiding construct and returned 354 complete questionnaires. Finally, the author distributed questionnaires regarding the mental health self-efficacy construct for the fourth time and received 310 complete and useable responses for data analyses. Hence, the empirical analyses of the present study are based on a 310 sample size.

### Measures

This study used five points Likert scale to measure the participants’ responses. This scale consists of five numbers where 1 means “strongly disagree,” 2 means “disagree,” 3 means “neutral,” 4 means “agree,” and 5 means “strongly agree.” This study considered previously validated items to assess the variables.

#### Interpersonal trust

The construct of interpersonal trust was measured with three items scale adopted from (Chen et al., 2018). The sample item included, “People take advantage of each other; if you do not be careful, you’ll suffer losses.” The Cronbach alpha value is 0.896.

#### Emotional exhaustion

The construct of emotional exhaustion was measured with seven items scale adopted from (Paulsen et al., 2005). The sample item included, “I feel fatigued when I get up in the morning and have to face another day in the class.” The Cronbach alpha value is 0.895.

#### Knowledge hiding

The construct knowledge hiding was measured with six items scale adopted from (Duan et al., 2022). The sample item included, “I pretended not to know or understand what he/she said, although I knew.” The Cronbach alpha value is 0.876.

#### Mental health self-efficacy

The construct mental health self-efficacy was measured with six items scale adopted from (Clarke et al., 2014). The sample item included, “I can keep my stress, anxiety or depression from interfering with the things that I want to do.” The Cronbach alpha value is 0.925.

## Results

### Assessment of measurement and structural model (mediation)

Structural equation modeling (SEM) is considered one of the most appropriate statistical models for data analyses. Structural equation modeling (SEM) consists of two different types, which include covariance-based (CB-SEM) and variance-based partial least squares structural equation modeling (PLS-SEM; Hair et al., 2019a). The main difference in both methods is that CB-SEM is considered for theory acceptance and rejection, while PLS-SEM is considered for advancing and developing the theories (Bashir et al., 2021). The present study applied the (PLS-SEM) technique for data analysis. The key rationale behind this selection is the usefulness of PLS-SEM for both confirmatory and exploratory studies (Hair et al., 2011). PLS-SEM is a useful approach for complex and multi-orders-based models and needs no specific data normality conditions. PLS-SEM is also suitable for evaluating small data sets (Hair et al., 2016). Hence, the present study considers the PLS-SEM method for empirical data analyses using Smart PLS 3.3.3 software. The outcomes of PLS-SEM-based analysis are evaluated in two stages, including model measurement and structural model evaluation. The measurement model stage assesses the reliability and validity of the constructs, whereas the structural model investigates the relationship between the proposed hypotheses. The acceptance or rejection of a hypothesis is determined through the “t” statistic and “p” values.

The model comprises 16 reflective items of three variables (Table 1). The results of model measurement consist of two parts: model reliability and validity. The present study considered the values of “Cronbach’s alpha, roh-A, composite reliability, and average variance extract (AVE)” to confirm the model’s reliability (Hair et al., 2019a), and all values are shown in Table 1. The values of Cronbach’s alpha are accepted if they are greater than 0.7 (Hair et al., 2019b). Similarly, composite reliability values are considered satisfactory if they exceed 0.7. Cronbach’s alpha values of models’ constructs (emotional exhaustion, interpersonal distrust, and knowledge hiding) are 0.895, 0.896, and 0.876, and the composite reliability values of models’ constructs are 0.918, 0.935, and 0.904, respectively. All values of Cronbach’s alpha and composite reliability are according to acceptable standards, which confirm the model’s reliability in the current study. The roh-A reliability values (0.897, 0.901, and 0.891) are also according to acceptable criteria (Hair et al., 2019b). The average variance extract (AVE) values exceeding 0.5 are considered appropriate for the model’s convergent validity (Hair et al., 2019a; Bashir et al., 2021). The Table 1 illustrates that the AVE values (0.615, 0.827, and 0.611) are according to acceptable criteria.

**Table 1:** Reliability and convergent validity of the study constructs (mediation).

Construct	Item	Outer loadings	VIF	Alpha	roh-A	Composite reliability	AVE
EEX	EEX1	0.824	2.456	0.895	0.897	0.918	0.615
	EEX2	0.749	1.983				
	EEX3	0.776	2.015				
	EEX4	0.770	2.047				
	EEX5	0.806	2.142				
	EEX6	0.774	2.067				
	EEX7	0.786	2.134				
IPDT	IPDT1	0.901	2.319	0.896	0.901	0.935	0.827
	IPDT2	0.907	2.975				
	IPDT3	0.920	3.253				
KH	KH1	0.769	2.505	0.876	0.891	0.904	0.611
	KH2	0.727	3.307				
	KH3	0.737	3.516				
	KH4	0.805	2.213				
	KH5	0.840	2.884				
	KH6	0.805	2.384				

EEX, emotional exhaustion; IPDT, interpersonal distrust; KH, knowledge hiding.

**Figure 2:**
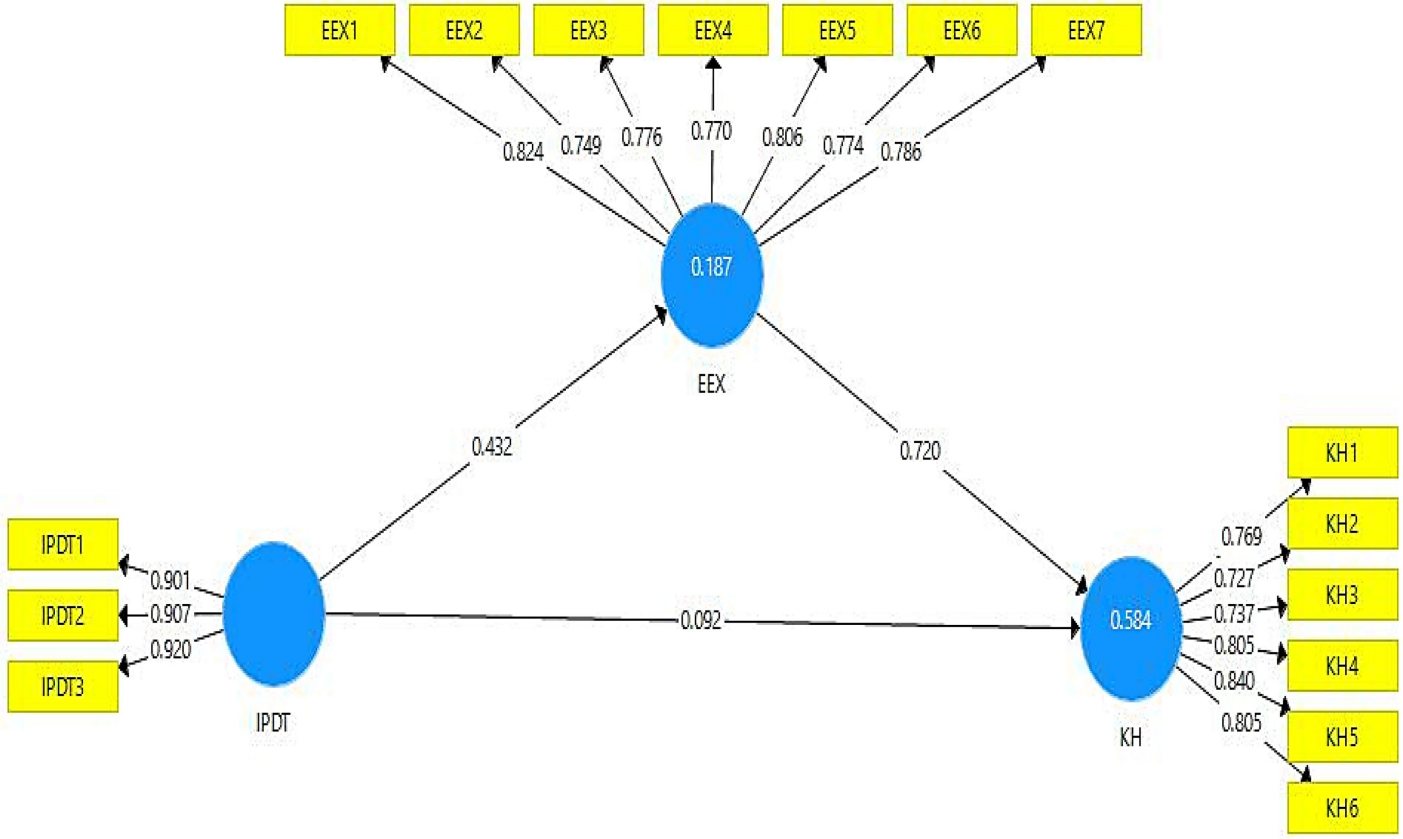
Path estimates (mediation).

All items’ outer loading values of models’ constructs are defined in Table 1. According to the required criteria, the outer loading values greater than or equal to 0.7 are considered reliable for the model’s validity (Hair et al., 2016). Figure 2 shows that the outer loading values of all constructs’ items are according to the accepted criteria. The variance inflation factor (VIF) values are also depicted in Table 1. The VIF values are assessed to validate the collinearity issues in the model. The model is considered free from collinearity issues if the VIF values are less than 0.5 (Hair et al., 2019b). According to the results in Table 1, all VIF values are less than 0.5, such as the variable “knowledge hiding” item KH-3 has the highest VIF value (3.516). Hence, it is proved that there are no collinearity issues in the present study model. The *R*^2^ values are considered to define the model’s strength. The values of latent variables greater than or near 0.5 indicate moderate strength of the model, and the values near or below 0.25 show weak model strength (Hair et al., 2014). The R^2^ value of the current study’s model’s endogenous variable (knowledge hiding) is 0.584, which shows moderate model strength. However, the value of the endogenous variable (emotional exhaustion) is 0.187, which shows the weak model strength. The model’s cross-validated redundancy (Q^2^) values are considered significant if they are larger than zero (Hair et al., 2019a). The *Q*^2^ values of all latent variables of the present study are greater than zero, demonstrating the model’s significance.

The two well-known approaches (Fornell–Larcker criterion and heterotrait–monotrait (HTMT) ratios) are used to approve the discriminant validity of the current study (Hair et al., 2016). The Fornell–Larcker criterion is assessed by taking the square roots of AVE values of the model’s variables (Hair et al., 2014). The Fornell–Larcker criterion values of variables are presented in Table 2. The values under the Fornell–Larcker criterion are accepted if the upper side first value of each column is highest than their below values. Table 2 demonstrates that all values of the Forenell–Larcker criterion are as per the required criteria. Thus, it is confirmed that discriminant validity based on the Fornell–Larcker criterion has been achieved in this study model. In addition, according to the given criteria, the HTMT values of all constructs should be less than 0.85; however, values greater than 0.90 are also acceptable (Hair et al., 2019b). According to the present study results, the HTMT values of constructs are less than 0.85, which confirms that discriminant validity in the present study’s model has been established (Table 3).

**Table 2:** Discriminant validity (Fornell-Larker-1981 criteria; mediation).

Construct	EEX	IPDT	KH
EEX	0.784		
IPDT	0.432	0.909	
KH	0.760	0.403	0.781

EEX, emotional exhaustion; IPDT, interpersonal distrust; KH, knowledge hiding. They shows the significance values and relationship between variables.

**Table 3:** Discriminant validity (HTMT; mediation).

Construct	EEX	IPDT	KH
EEX	–	–	–
IPDT	0.475	–	–
KH	0.825	0.449	–

EEX, emotional exhaustion; IPDT, interpersonal distrust; KH, knowledge hiding.

### Model estimation, direct and indirect (mediation)

The current study’s empirical examination uses a bootstrapping approach through 5,000 samples with replacements to estimate the significance level. The direct, indirect, and total paths are depicted in Table 4. The present study considered the “*t*” values and “*p*” values of statistics for the acceptance or rejection of proposed hypotheses. The current study hypothesis results are shown in Table 5. According to hypothesis 1, interpersonal distrust positively correlates with knowledge hiding; however, the outcomes (*t* = 1.886, *p* = 0.059) depicted that H1 of the present study is not accepted. The outcomes (*t* = 5.418, *p* = 0.000) of H2 confirmed that interpersonal distrust positively enhances emotional exhaustion, which means the second hypothesis of the present study is accepted. Additionally, the beta value of H2 revealed that one unit change in the independent variable (interpersonal distrust) would result in 0.432 changes in the dependent variable (emotional exhaustion).

**Table 4:** Direct, indirect and total path estimates (mediation).

	Beta	SD	*t*	*p*
Direct path				
EEX –> KH	0.720	0.051	14.230	0.000
IPDT –> EEX	0.432	0.080	5.418	0.000
IPDT –> KH	0.092	0.049	1.886	0.059
Indirect path				
IPDT –> EEX –> KH	0.311	0.071	4.397	0.000
Total path				
EEX –> KH	0.720	0.051	14.230	0.000
IPDT –> EEX	0.432	0.080	5.418	0.000
IPDT –> KH	0.403	0.077	5.221	0.000

EEX, emotional exhaustion; IPDT, interpersonal distrust; KH, knowledge hiding. They shows the significance values and relationship between variables.

**Table 5:** Hypotheses testing (mediation).

		Coefficient (Beta)	SD	*t*	*p*	Status
Hypotheses						
H1	IPDT –> KH	0.092	0.049	1.886	0.059	Not supported
H2	IPDT –> EEX	0.432	0.080	5.418	0.000	Supported
H3	EEX –> KH	0.720	0.051	14.230	0.000	Supported
Mediation hypotheses						
H4	IPDT –> EEX –> KH	0.311	0.071	4.397	0.000	Supported

EEX, emotional exhaustion; IPDT, interpersonal distrust; KH, knowledge hiding.

According to the results (*t* = 14.230, *p* = 0.000) of the third hypothesis, emotional exhaustion positively enhances knowledge hiding, which confirms that the H3 of the present study is also accepted. In addition, the beta value of the third hypothesis showed that one unit change in the independent variable (emotional exhaustion) would result in 0.720 changes in the dependent variable (knowledge hiding). The present study also considered the mediating role of emotional exhaustion in the relationship between interpersonal distrust and knowledge hiding. For the empirical investigation of emotional exhaustion as a mediator, this study assumes H4 (emotional exhaustion positively mediates the relationship between interpersonal distrust and knowledge hiding.). Results of H4 (*t* = 4.397, *p* = 0.000) confirm that emotional exhaustion positively mediates the relationship between interpersonal distrust and knowledge hiding. The path value (0.311) of H4 also confirms that the hypothesis is accepted.

### Assessment of measurement and structural model (moderation analysis)

For a reflective measurement of the model, Smart-PLS recommends a two-stage method for moderation analysis, including “model measurement and model estimation” (Hair et al., 2016). The moderation analysis of the current study defines that all basic criteria (construct reliability and validity) and indicators of model assessment such as out loading values, CR, Cronbach’s alpha, rho_A, and AVE are according to acceptable criteria (Hair et al., 2016). Table 6 describes the particulars of model assessment indicators.

**Table 6:** Reliability and convergent validity of the study constructs (moderation).

Construct	Item	Outer loadings	VIF	Alpha	roh-A	Composite reliability	AVE
EEX	EEX1	0.824	2.456	0.895	0.897	0.918	0.615
	EEX2	0.749	1.983				
	EEX3	0.776	2.015				
	EEX4	0.770	2.047				
	EEX5	0.806	2.142				
	EEX6	0.774	2.067				
	EEX7	0.786	2.134				
IPDT	IPDT1	0.901	2.319	0.896	0.901	0.935	0.827
	IPDT2	0.907	2.975				
	IPDT3	0.920	3.253				
KH	KH1	0.769	2.505	0.876	0.891	0.904	0.611
	KH2	0.727	3.307				
	KH3	0.737	3.516				
	KH4	0.805	2.213				
	KH5	0.840	2.884				
	KH6	0.805	2.384				
MHSE	MHSE1	0.831	2.598	0.925	0.926	0.941	0.728
	MHSE2	0.871	3.174				
	MHSE3	0.883	3.365				
	MHSE4	0.848	2.635				
	MHSE5	0.873	3.023				
	MHSE6	0.809	2.215				

EEX, emotional exhaustion; IPDT, interpersonal distrust; KH, knowledge hiding.

The results of moderation analysis confirmed the discriminant validity with moderation effect (mental health self-efficacy) through two approaches (Fornell–Larcker criterion and HTMT ratios). Tables 7, 8 describe the Fornell–Larcker criterion and HTMT ratios, respectively. The results also explain that the inner VIF values of all variables are significantly lower than 5 (Table 6), which approves that there is no collinearity issue in the present study data. The *R*^2^ values of endogenous variables of the current study’s model (emotional exhaustion and knowledge hiding) are 0.187 and 0.591, which shows moderate and weak model strength, respectively (Hair et al., 2019a).

**Table 7:** Discriminant validity (Fornell-Larker-1981 criteria; moderation).

Construct	EEX	IPTD	KH	MHSE	MHSE*EEX
EEX	0.784				
IPTD	0.432	0.909			
KH	0.760	0.403	0.781		
MHSE	0.697	0.366	0.543	0.853	
MHSE*EEX	−0.741	−0.458	−0.629	−0.501	1.000

EEX, emotional exhaustion; IPDT, interpersonal distrust; KH, knowledge hiding. They shows the significance values and relationship between variables.

**Table 8:** Discriminant validity (HTMT; moderation).

Construct	EEX	IPTD	KH	MHSE	MHSE*EEX
EEX	–	–	–	–	–
IPTD	0.475	–	–	–	–
KH	0.825	0.449	–	–	–
MHSE	0.763	0.399	0.583	–	–
MHSE*EEX	0.784	0.483	0.646	0.520	–

EEX, emotional exhaustion; IPDT, interpersonal distrust; KH, knowledge hiding.

### Model estimation (moderation)

**Figure 3:**
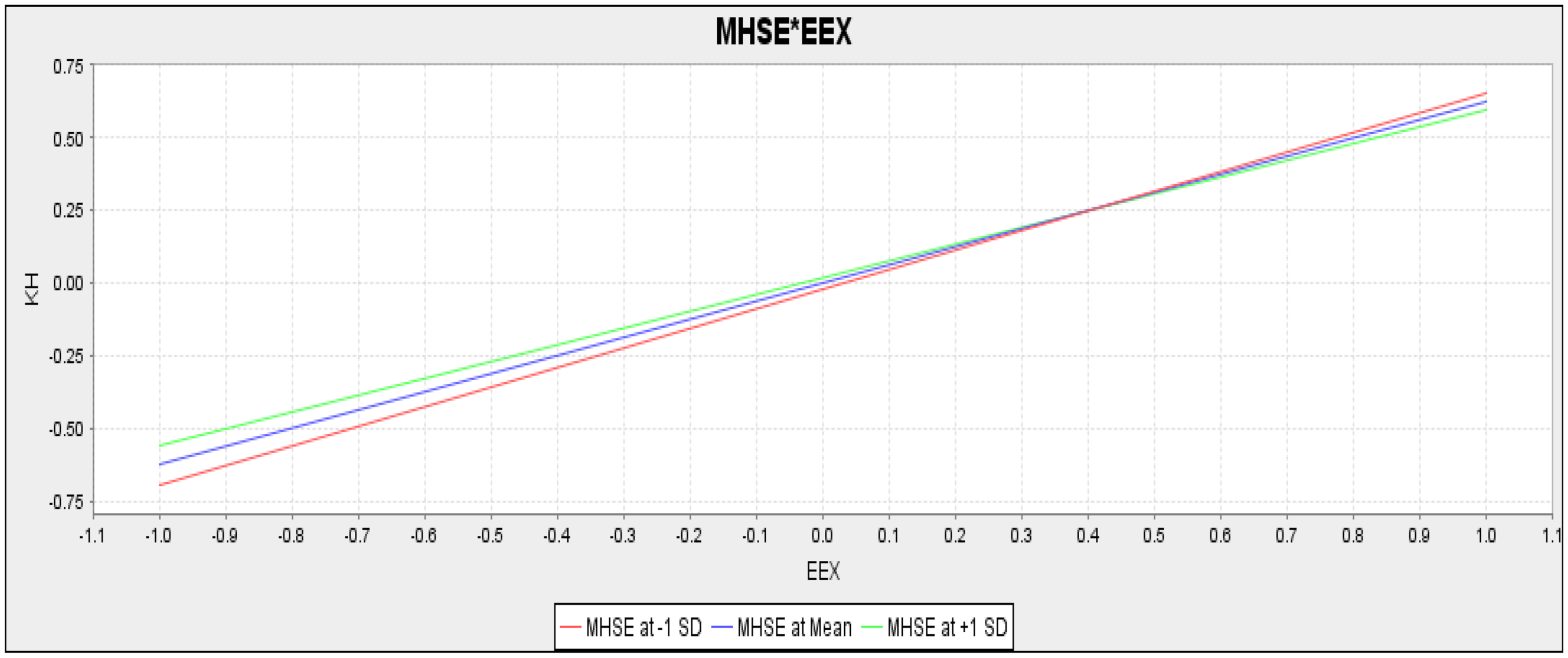
Slope for mental health self-efficacy (MHSE) and emotional exhaustion (EEX; moderation).

The present study also checked the moderating role of mental health self-efficacy in the relationship between emotional exhaustion and knowledge hiding. For empirical investigation, the current study assumes H5 as mental health self-efficacy negatively moderates the relationship between emotional exhaustion and knowledge hiding. The results (*t* = 2.580, *p* = 0.010) confirmed that mental health self-efficacy negatively moderates the relationship between emotional exhaustion and knowledge hiding. Hence H5 of the present study is accepted (Table 9). Mental health self-efficacy negatively moderates the slope for the relationship between emotional exhaustion and knowledge hiding. The slope is given in Figure 3.

**Table 9:** Hypotheses testing (moderation).

	Moderation hypotheses	Coefficient (Beta)	SD	*t*	*p*	Status
H5	MHSE*EEX –> KH	−0.048	0.019	2.580	0.010	Supported

EEX, emotional exhaustion; KH, knowledge hiding; MHSE, mental health self-efficacy.

## Discussion

The present era is knowledge-intensive, where knowledge is considered a power (Duan et al., 2022). Firms now realize that the knowledge and skills of employees could play a valuable role in organizational success. However, the knowledge-hiding behavior of individuals is a hurdle in the transfer of knowledge and expertise. It becomes more critical in the case of teaching institutions, where the students are taught and trained. Scholars are now giving attention to pursuing the antecedents and consequences of knowledge-hiding behavior. This study also attempts to determine the role of interpersonal distrust as an antecedent of knowledge hiding behavior of music education students. Based on the social exchange theory, this study tries to check the association of interpersonal distrust with emotional exhaustion and knowledge hiding.

For empirical investigation, this study proposes five hypotheses. First, this study assumes that interpersonal distrust positively enhances knowledge hiding. According to the second hypothesis, interpersonal distrust positively enhances emotional exhaustion. Third, the current study assumes that emotional exhaustion is positively associated with knowledge hiding. For determining the mediating role, this study proposes that emotional exhaustion positively mediates the relationship between interpersonal distrust and knowledge hiding. This study also attempts to check the moderating role of mental health self-efficacy in the relationship between emotional exhaustion and knowledge hiding. The findings of this study revealed that interpersonal distrust does not directly influence knowledge hiding, which means the first hypothesis of this study is rejected. However, these outcomes are inconsistent with prior studies (Connelly et al., 2012; Yuan et al., 2020; Jafari-Sadeghi et al., 2022). According to these studies, when individuals did not trust each other due to personal conflicts or personality clashes, it would adversely impact their commitment and citizenship behavior. Moreover, the scholars also noticed that interpersonal distrust might create negative feelings among individuals, and in return, their knowledge-sharing behavior is also influenced negatively. The revealing of this study further acknowledged that interpersonal distrust positively enhances emotional exhaustion, which means the second hypothesis of this study is accepted. These findings are consistent with previous studies (Martínez-Iñigo et al., 2018; Said et al., 2021). According to these scholars, when individuals experience interpersonal distress, their mental and emotional health effects adversely. Moreover, the individuals feel emotionally exhausted when facing conflicts and personality clashes with others. The students also face difficulties completing their academic tasks when they experience interpersonal conflicts with their teachers. According to the present study’s findings, the third hypothesis is also accepted, which means that emotional exhaustion leads to knowledge hiding. These findings have similarities with existing studies (Yao et al., 2020; Ain et al., 2022). According to these scholars, when individuals experience emotionally exhausted, they may adopt negative behavior in the workplace. In return, they may hide their knowledge and expertise to decrease their stress and frustration. Additionally, Guo et al. (2021) noticed that emotionally exhausted people might hide information in order to save their depleted resources. Moreover, they stated that knowledge concealing might be used in knowledge demands as a defensive tactic to deal with emotional depletion. People who experience high degrees of emotional exhaustion have little remaining emotional and physical energy.

The present study also assessed the mediating role of emotional exhaustion in the relationship between interpersonal distrust and knowledge hiding. The results confirmed that the fourth hypothesis is accepted, which means that emotional exhaustion positively mediates the relationship between interpersonal distrust and knowledge hiding. These results have consistency with previous studies (Zhao and Jiang, 2021; Ain et al., 2022). These studies acknowledged that emotional exhaustion leads employees to develop the undesired behavior of knowledge hiding. Venz and Nesher Shoshan (2022) noticed that role stress, as an imbalance in interpersonal connection, may cause emotional weariness in workers. This unpleasant emotional experience may then result in either direct or indirect emotion-driven behaviors as a means of expressing their passive sentiments. Moreover, when employees are emotionally exhausted, they may purposefully fail to assist their coworkers (Zhao and Jiang, 2021). The present study also assumes the moderating role of mental health self-efficacy in the relationship between emotional exhaustion and knowledge hiding. The findings acknowledged that mental health self-efficacy negatively moderates the relationship between emotional exhaustion and knowledge hiding, which means the fifth hypothesis of this study is accepted. These findings have consistency with prior studies (Gago-Valiente et al., 2021; Martínez-Líbano et al., 2021). According to these studies, individuals’ mental health is adversely impacted when they feel emotionally exhausted and distress.

## Theoretical and practical implications

One of the main objectives of this research was to explain the significant theoretical implications related to mental health self-efficacy and its mediating role between knowledge hiding and emotional exhaustion. It is important to mention that no earlier study has discussed this aspect of interpersonal distrust and its relationship with knowledge hiding among music students. It is crucial to study these factors to explore the behavioral status of the students which can affect their performance and mental health status. The current study extends the available literature on mental health efficacy, knowledge hiding, emotional exhaustion and interpersonal distrust. It also adds to the theoretical literature *via* novel data analysis and novel findings of research done on music education students. The study has explored the mediating role of mental health self-efficacy which can be used as a tool to mediate the relationship between interpersonal distrust, emotional exhaustion and knowledge hiding.

Many studies have been conducted on moderating the role of self-efficacy but no study has used these variables along with mental health self-efficacy to conduct research on music students which makes this research study a novel and unique one. This study fills the theoretical gap in the literature by adding valuable literature *via* literature review and novel data analysis. The study finds that interpersonal distrust positively enhances knowledge hiding and emotional exhaustion. It has also explored that emotional exhaustion positively enhances knowledge hiding as well as acts as a moderator between interpersonal distrust and knowledge hiding. The study has also explored the mediating role of mental health self-efficacy. The results of this study can be used by future scientists and researchers as a reference to plan new research models in the relevant area of research. Educationists and research scientists can plan the use of mental health self-efficacy to enhance the performance of students.

Along with theoretical implications the current study has various, relevant and genuine practical implications. First of all, this study increases knowledge and awareness about the negative effects of knowledge hiding, emotional exhaustion and their effect on the creativity and performance of students. It also throws light on the mediating role of mental health self-efficacy between interpersonal distrust, knowledge hiding and emotional exhaustion. Thus, all the stakeholders can use the findings of this study to control these negative behaviors to make the organizational environment more productive and positive. To mitigate the negative events, the practice of mental health efficacy can be strengthened by teachers and managers. This strengthening of mental health efficacy will boost interpersonal trust and knowledge sharing among colleagues. Awareness and training programs, counseling sessions, and improving communication and modeling techniques can be used to obtain the desired outcomes.

## Limitations

Like other social sciences studies, the present study also has some limitations, which may become opportunities for scholars to conduct their research in the future. First, this study is conducted using a small sample size; in the future, researchers may broaden the sample size to verify the present study’s model. Second, this study collected the data using a structured questionnaire method; in the future, scholars may consider other data collection methods such as semi-structured questionnaires, interview methods, etc. Third, this study Is conducted in China, and the results may not be generalizable to other contexts. Scholars in the future may conduct the same study in other developing or developed countries for a better understanding of the study model. Fourth, the present study assumes the interpersonal distrust as an antecedent of knowledge hiding; future studies may consider other possible antecedents of knowledge hiding like workplace ostracism and psychological contract violation. Fifth, this study examined the mediating role of emotional exhaustion in the relationship between interpersonal distrust and knowledge hiding; future studies may consider other possible mediating variables like cynicism and burnout, etc. Finally, this study assumes moderating role of mental health self-efficacy in the relationship between emotional exhaustion and knowledge hiding; future researchers may consider other moderators for conducting this study, like emotional intelligence and commitment.

## Conclusion

The knowledge-hiding behavior of individuals is a hurdle in the internal dissemination of knowledge and expertise. It becomes more critical in the case of teaching institutions, where the students are taught and trained. Scholars are now seeking ways to deal with the knowledge-hiding phenomenon and working on the antecedents and consequences of knowledge-hiding behavior. This study also attempts to determine the role of interpersonal distrust as an antecedent of knowledge hiding behavior of music education students. Based on the social exchange theory, this study assumes that interpersonal distrust positively enhances knowledge hiding and emotional exhaustion. The present study also attempts to check the association of emotional exhaustion with knowledge hiding. Evaluating the mediating role of emotional exhaustion in the relationship between interpersonal distrust and knowledge hiding is also an important objective of the present study. This current study also aims to check the moderating role of mental health self-efficacy in the relationship between emotional exhaustion and knowledge hiding. The findings of this study revealed that interpersonal distrust does not directly influence knowledge hiding; however, interpersonal distrust has a positive association with emotional exhaustion. The findings also depicted that emotional exhaustion positively correlates with knowledge hiding. The results also confirmed that emotional exhaustion positively mediates the relationship between interpersonal distrust and knowledge hiding. Further, the outcomes acknowledged that mental health self-efficacy negatively moderates the relationship between emotional exhaustion and knowledge hiding.

## Data availability statement

The original contributions presented in the study are included in the article/supplementary material, further inquiries can be directed to the corresponding author.

## Ethical statement

The studies involving human participants were reviewed and approved by Xihua University, China. The patients/participants provided their written informed consent to participate in this study. The study was conducted in accordance with the Declaration of Helsinki.

## Author contributions

XZ: conceptualization, data collection, and writing the draft. The author agreed to the submitted version of manuscript.

## Conflict of interest

The author declares that the research was conducted in the absence of any commercial or financial relationships that could be construed as a potential conflict of interest.

## Publisher’s note

All claims expressed in this article are solely those of the authors and do not necessarily represent those of their affiliated organizations, or those of the publisher, the editors and the reviewers. Any product that may be evaluated in this article, or claim that may be made by its manufacturer, is not guaranteed or endorsed by the publisher.
